# Age and CHADS_2_ Score Predict the Effectiveness of Renin-Angiotensin System Blockers on Primary Prevention of Atrial Fibrillation

**DOI:** 10.1038/srep11442

**Published:** 2015-06-22

**Authors:** Chen-Ying Hung, Yu-Cheng Hsieh, Cheng-Hung Li, Jin-Long Huang, Ching-Heng Lin, Tsu-Juey Wu

**Affiliations:** 1 Cardiovascular Center, Taichung Veterans General Hospital, Taichung, Taiwan; 2 Department of Internal Medicine, Taipei Veterans General Hospital, Hsinchu Branch, Hsinchu, Taiwan; 3 Department of Nutrition, Hungkuang University, Taichung, Taiwan; 4 Department of Internal Medicine, Faculty of Medicine, Institute of Clinical Medicine, Cardiovascular Research Center, National Yang-Ming University School of Medicine, Taipei, Taiwan; 5 School of Medicine, Chung Shan Medical University, Taichung, Taiwan; 6 Department of Medical Research, Taichung Veterans General Hospital, Taichung, Taiwan

## Abstract

Renin-angiotensin system (RAS) blockers have potential protective effects against atrial fibrillation (AF). The purpose of this study was to determine if patient characteristics and underlying co-morbidities could predict the efficacy of RAS blockers in AF prevention. Patients aged ≥ 45 years with hypertension were identified from the Taiwan National Health Insurance Research Database. After propensity-score matching, a total of 22,324 patients were included in this study. Risk of new-onset AF in RAS blockers users and non-users was estimated. During up to 10 years of follow-up, 1,475 patients experienced new-onset AF. Overall, RAS blockers reduced the risk of AF by 36% (adjusted HR 0.64; 95% CI 0.58 to 0.71; p < 0.001). Subgroup analysis showed that RAS blockers use was beneficial for AF prevention in patients aged ≥ 55 years or with a CHADS_2_ score of 1, 2, or 3. The therapy provided no obvious beneficial effect for AF prevention in those aged less than 55 years or with a CHADS_2_ score ≥ 4. In conclusion, RAS blockers reduced the risk of new-onset AF in patients aged ≥ 55 years or with a CHADS_2_ score of 1, 2, or 3, but not in patients aged less than 55 years or with a CHADS_2_ score ≥ 4.

Atrial fibrillation (AF) is the most common arrhythmia and is associated with an increased risk of stroke, mortality, and health care costs^1^,^2^. Old age, male gender, hypertension, heart failure, diabetes mellitus, vascular disease, pulmonary disease, thyroid disease, chronic renal disease, and valvular heart disease are risk factors for AF occurrence^2^,^3^,^4^,^5^. Among these risk factors, hypertension is the most common condition and is associated with a 40–50% increased risk of developing new-onset AF^3^. As the elderly population increased in recent years, AF has become more prevalent. Therefore, a major focus of disease management is to effectively prevent new-onset AF in hypertensive patients^1^.

The focus of AF primary prevention in patients with hypertension has recently shifted to renin-angiotensin system (RAS) blockers; such agents include angiotensin-converting enzyme inhibitors (ACEIs) and angiotensin-receptor blockers (ARBs)^6^,^7^. Studies have suggested that RAS blockers have favorable potency because of their effect against atrial remodeling^8^,^9^. The key targets of these therapies are electrical and structural changes in the atria, such as inflammation, hypertrophy, and oxidative stress^6^. Although some large randomized trials^10^,^11^ and nationwide retrospective studies^7^,^12^ have shown that RAS blockers can reduce the risk of new-onset AF in patients with significant structural heart disease, the evidence is less robust in hypertensive patients with mild-to-moderate heart disease^6^,^13^.

Since trials investigating the effect of RAS blockers on AF prevention in hypertensive patients without significant heart disease reported conflicting results,^7^ whether these therapies can prevent AF in these subgroups of patients remains a subject of debate. Our recent studies suggest that CHADS_2_ scores could be used for predicting the AF preventive effect of statin, another upstream therapy for AF prevention^14^,^15^,^16^. However, it is still unclear whether this co-morbidity score can be used to predict RAS blockers effects on AF prevention. The purpose of the present study was to determine if patient characteristics or cardiovascular co-morbidity scoring systems could predict the effectiveness of RAS blockers in primary AF prevention in a nationwide population-based cohort.

## Methods

### Study database

The National Health Insurance program, which covers about 99% of population and all forms of health care services in Taiwan, was implemented in 1995. The National Health Research Institute (NHRI) of Taiwan has established a National Health Insurance Research Database. In the present study, we used the Longitudinal Health Insurance Database 2000, a systemic sampling of patient data released by the NHRI, which includes a total of 1,000,000 subjects. The NHRI has confirmed these random samples to be representative of the general Taiwanese population; i.e., there were no statistically significant differences in age and gender between overall population and the sample.

Patients’ demographic characteristics were included in the database. These data also contain information about prescriptions, including the drug use duration and prescribed dosage. The information about diagnoses and prescriptions is of high quality, and has previously been used for several epidemiological studies^17^,^18^. The NHRI safeguards the privacy of individuals and provides the data to researchers after ethical approval has been obtained. The NHRI made data at the individual level available to us in an anonymous format, in which specific individuals cannot be identified. This study was approved by the Institutional Review Board of Taichung Veterans General Hospital (CE14148).

### Study population

**Figure 1:**
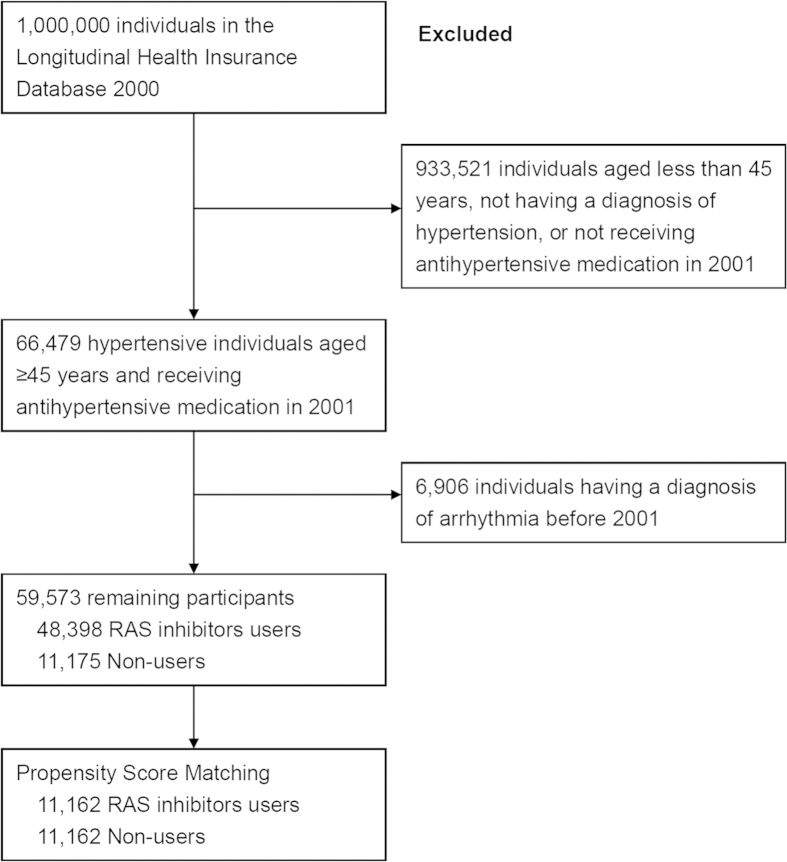
Inclusion and exclusion criteria of study participants.

We identified individuals with a hypertension diagnosis and receiving anti-hypertensive therapy in 2000 and 2001. We excluded all individuals suffering from AF or other arrhythmia. We matched patients on RAS blockers 1:1 with individuals using other antihypertensives. Each matched cohort was followed from January 1, 2002 until a diagnosis of AF, or end of follow-up on December 31, 2011, whichever came first. The propensity-score matching was performed in an attempt to address differences in medical history between users as previously described^19^. Factors used in the propensity-score matching were age, gender, co-morbidities, and Charlson co-morbidity index. Inclusion and exclusion criteria of study participants are shown in Fig. 1.

### Study endpoint

The study endpoint was defined as new-onset AF (International Classification of Diseases, Ninth Revision, Clinical Modification [ICD-9-CM] code 427.31) during the 10-year follow-up period (2002-2011). All occurrences of AF were confirmed by the claims data. To ensure the diagnostic validity, only patients with an inpatient AF diagnosis or at least 3 consensus AF diagnoses at outpatient departments (to avoid misclassification by including patients with tentative diagnosis for exams and those retrieving exam reports) were included. The date of the end-point event (AF) was defined as the index date.

### Medications and co-morbidities

RAS blockers (ACEIs and ARBs) use records were retrieved from outpatient and inpatient claims database. The prescription dates and the number of pills per prescription were collected. Patients were divided into RAS blockers users group and non-users group according to their ACEIs and ARBs use between January 1, 2001, and the index date (if AF occurred), or December 31, 2011. Patients who used ACEIs or ARBs for more than 28 days were defined as RAS blockers users. Other medications were identified between January 1, 2000, and December 31, 2001.

We identified many cardiovascular co-morbidities as a potential confounders by using ICD-9-CM code between January 1, 2000, and December, 31, 2001. The CHADS_2_ score was calculated for each patient by assigning 1 point each for the presence of hypertension, heart failure, age ≥ 75 years, and diabetes, and 2 points for a history of stroke or transient ischemic attack (TIA)^20^. The CHA_2_DS_2_VASc score was calculated for each patient by assigning 1 point each for the presence of hypertension, heart failure, age 65–74 years, diabetes, vascular disease, and female gender, and 2 points for a history of stroke or TIA, and age ≥ 75 years^21^.

### Statistical analysis

All analyses were performed on the propensity score 1:1 matched cohorts. We matched RAS blockers users and non-users for age, gender and co-morbidities; paired matching was included in the model as a stratification variable. The data are presented as the mean values and standard deviations (SD) for continuous variables, and proportions for categorical variables. The t test was used to analyze differences between continuous variables, and chi-square test was used to analyze differences between categorical variables. Multivariate Cox proportional hazard regression was used to estimate the hazard ratio (HR) and 95% confidence interval (CI), which was used to determine the association between the use of RAS blockers and the occurrence of AF. The AF-free survival curves were plotted via the Kaplan–Meier method with statistical significance examined by the log-rank test. All statistical analyses were performed by SAS software version 9.2 (SAS Institute, Inc., Cary, NC, USA). A p value of <0.05 was considered statistically significant.

## Results

Baseline characteristics of the RAS blockers users and non-users are shown in Table 1. A total of 22,324 patients aged ≥ 45 years were enrolled in this study after matching, of whom 11,162 (50%) had used RAS blockers. The mean age of the study population was 65.5 ± 11.3 years, with 30.4% of them aged 65–74 years, and 22.3% of them aged ≥ 75 years. Females accounted for 51.5% of the population. Among the study subjects, 4.8% had heart failure, 10.8% had diabetes mellitus, 10.5% had stroke or TIA, 1.9% had vascular disease, 1.3% had thyroid disease, 1.1% had valvular heart disease, 10.6% had chronic lung disease, and 2.5% had chronic renal disease. There was no significant difference in the distribution of age, gender and co-morbidities between groups. The rate of statin, aspirin, alpha-blocker, beta-blocker, calcium channel blocker, diuretics, and digoxin usage was higher among RAS blockers users than non-users (p = 0.022 for digoxin; p < 0.001 for other agents). The average CHADS_2_ score was 1.6 ± 0.9 and the average CHA_2_DS_2_VASc score was 2.6 ± 1.3.

### Treatment outcome

During up to 10 years of follow-up (a total of 177,798 person-years), 1,475 patients (6.6% of the study population) developed new-onset AF, and the overall incidence rate was 8.3 per 1,000 person-years. Table 2 shows the HRs for the development of new-onset AF in the cohort. AF occurred less frequently among RAS blockers users compared with non-users before and after an adjustment for variables between the 2 cohorts (adjusted HR 0.64; 95% CI 0.58 to 0.71). The incidence rate of AF decreased from 9.6 to 7.2 per 1,000 person-years after RAS blockers use. The adjusted HRs were 0.80, 0.64, and 0.77 for pure ACEIs users, pure ARBs users, and mixed users, respectively. ACEIs and ARBs therapy were both associated with lower incidences of new-onset AF compared with other agents (p = 0.007 for pure ACEIs users, p < 0.001 for pure ARBs users). Table 2 also shows the associations between the duration of RAS blockers use and the risk of AF. Among the RAS blockers users, 23.5% used RAS blockers for 28-365 days, and 76.5% of them used RAS blockers for >365 days. The incidence rates of AF were 9.6, 8.8, and 6.8 per 1,000 person-years among patients who used RAS blockers less than 28, 28 to 365, and more than 365 days, respectively. The occurrence of new-onset AF was significantly different between non-users and those who used RAS blockers for 28-365 days (adjusted HR 0.77; 95% CI 0.65 to 0.90) as well as those who used RAS blockers for >365 days (adjusted HR 0.60; 95% CI 0.54 to 0.67).

### Age, CHADS_2_ score, CHA_2_DS_2_VASc score and treatment outcome

**Figure 2:**
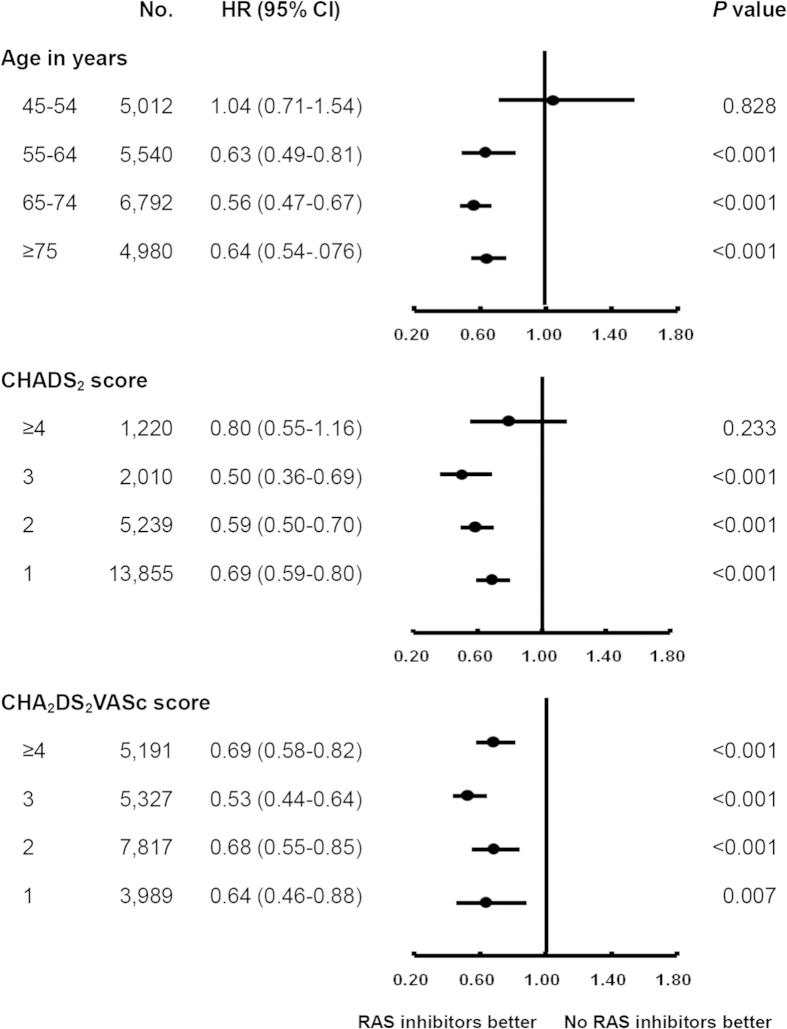
The effectiveness of RAS blockers against new-onset AF according to age, CHADS_2_ score, and CHA_2_DS_2_VASc score.

**Figure 3:**
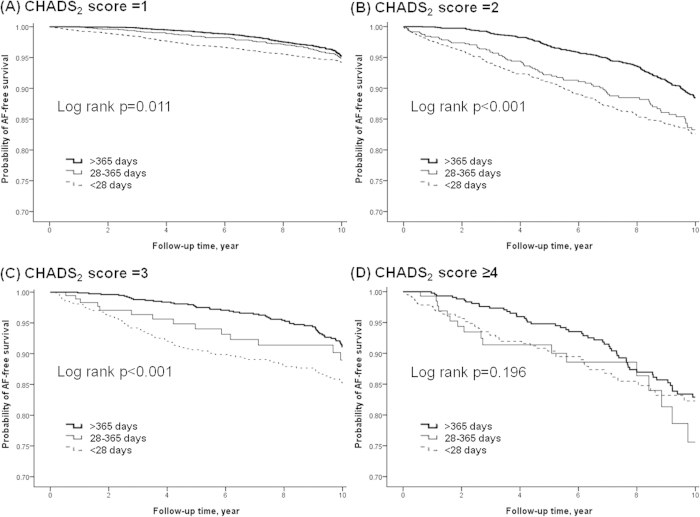
AF-free survival rate according to RAS blockers use and CHADS_2_ score.

In subgroup analyses, there was a universal RAS blockers protective effect across gender categories (For female: adjusted HR 0.73; 95% CI 0.63–0.84. For male: adjusted HR 0.56; 95% CI 0.48–0.65). Figure 2 displays the hazard ratio plot of the protective effect of RAS blockers against AF according to age, CHADS_2_ score, and CHA_2_DS_2_VASc score. RAS blockers use was beneficial for AF prevention in patients aged ≥55 years (For age 55–64: adjusted HR 0.63; 95% CI 0.49–0.81. For age 65–74: adjusted HR 0.56; 95% CI 0.47–0.67. For age ≥ 75: adjusted HR 0.64; 95% CI 0.54–0.76), but provided no obvious beneficial effect in those aged less than 55 years (adjusted HR 1.04; 95% CI 0.71–1.54). By using established cardiovascular co-morbidity scoring systems, RAS blockers use was beneficial in patients with a CHADS_2_ score of 1, 2, or 3 (For a score of 1: adjusted HR 0.69; 95% CI 0.59–0.80. For a score of 2: adjusted HR 0.59; 95% CI 0.50–0.70. For a score of 3: adjusted HR 0.50; 95% CI 0.36–0.69). No significant benefit was found in patients with a CHADS_2_ score ≥ 4 (adjusted HR 0.80; 95% CI 0.55 to 1.16). RAS blockers therapy shows similar efficacy across every CHA_2_DS_2_VASc score category. The Kaplan-Meier survival plot presented in Figure 3 shows the protective effect of RAS blockers against AF according to RAS blockers use and CHADS_2_ score. The survival curves began to diverge early and continued to diverge throughout the course of the study in patients with a CHADS_2_ score of 1, 2, and 3. In patients with a CHADS_2_ score ≥ 4, the treatment showed no significant effect (log rank p = 0.196).

## Discussion

The main finding of this nationwide cohort study is that the risk of AF was lower in RAS blockers users than non-users among patients aged ≥55 years, but not among patients aged less than 55 years. This study also showed that RAS blockers therapy reduces the risk of new-onset AF for those with a CHADS_2_ score of 1, 2, or 3. To the best of our knowledge, this is the first study to analyze the relationship between CHADS_2_ score and the protective effects of RAS blockers against AF. The results suggest that the CHADS_2_ score can help identify the patients who will benefit most from RAS blockers use for the prevention of AF. These findings support the inference that RAS blockers have anti-arrhythmic effects and explain the heterogeneity among previous trials.

Our results showed that RAS blockers reduce the risk of new-onset AF in patients aged ≥ 55 years. Of note, this therapy provided no obvious beneficial effect in those aged less than 55 years. AF is a result of electrical and structural remodeling of the atria, which involves progression of underlying heart disease, genetic factors, and ageing process. RAS blockers target both the formation and evolution of the substrate for AF, and thus prevent the occurrence of new-onset AF. Young patients have a low incidence of developing AF and fewer processes of atrial remodeling than elderly patients^22^. Therefore, our results may imply that RAS blockers do not play a major role in AF prevention in young patients. These findings may also explain why previous studies reached different conclusions^23^– heterogeneity of patient characteristics may confound effectiveness of RAS blockers against AF.

Our study also showed that RAS blockers are more beneficial in hypertensive patients with a CHADS_2_ score of 1, 2, or 3. Patients with scores ≥ 4 get no obvious AF preventive benefits from RAS blockers use. Meanwhile, grouping patients by using CHA_2_DS_2_VASc score showed a universal protective effect of this therapy. Therefore, the CHADS_2_ score is a more useful scoring system than the CHA_2_DS_2_VASc score for predicting the effectiveness of RAS blockers in AF prevention. The CHADS_2_ score is a convenient scoring system used for evaluating the complexity of patient’s co-morbidities. Previous study demonstrates that high CHADS_2_ scores are associated with atrial remodeling and a larger left atrium size^24^,^25^. Furthermore, patients with higher CHADS_2_ scores had a significantly larger amount of atrial fibrosis and advanced atrial remodeling when compared to those with lower CHADS_2_ scores^26^,^27^. We propose that atrial fibrosis and fixed atrial remodeling may therefore reduce the potency of RAS blockers in patient with high CHADS_2_ scores.

Although studies focusing on the protective effects of RAS blockers against AF have yielded consistently significant results in people with heart failure, these effects are less clear in patients with multiple risk factors, such as hypertension, diabetes mellitus, hypercholesterolemia, and coronary artery disease^6^. A recent meta-analysis, which included patients at high cardiovascular risk, showed a 19% risk reduction in the occurrence of AF with RAS blockers^28^. However, heterogeneity among studies was noted, especially in subjects with mild-to-moderate underlying heart disease. Hence, whether a specific population of hypertensive patients may benefit from RAS blockers in preventing AF is the question addressed in the present study. Our results suggest that RAS blockers reduce the risk of new-onset AF in patients with cardiovascular diseases, and the effect was confound by age and CHADS_2_ score. This finding provides an explanation for the heterogeneity among studies.

Our previous studies revealed that statin was more beneficial in patients with higher CHADS_2_ scores than those with lower scores for AF prevention^15^. In the present study, RAS blockers seemed to have a different pattern of efficacy for AF prevention in regard to this co-morbidity score: RAS blockers were less effective in patients with high CHADS_2_ score. The result of our study implies that the main mechanisms of AF prevention by statin and RAS blockers may be different. RAS blockers can reduce the occurrence of new-onset AF via counteracting the profibrotic process, but not fixed atrial fibrosis^6^. On the other hand, anti-inflammatory effect of statin may be more obvious in regarding to AF prevention^16^. Patients with higher CHADS_2_ scores may have a more severe inflammation status and a more advanced fixed atrial fibrosis than those with lower scores. This difference may therefore result in the different efficacy pattern of stain and RAS blockers.

Our study has a number of strengths. The data on this study cohort was retrieved from a computerized population-based database, allowing evaluation of a large cohort in a 10-year follow-up period. In addition, because the data on RAS blockers use includes all prescription information before the diagnosis of AF, recall bias can be avoided. Furthermore, we conducted multivariate analysis to eliminate the misclassifications, and the results revealed no significant changes in the HRs before and after the analyses. Finally, we matched individuals on a propensity score to address differences in medical history between groups. This method further reduces the likelihood of bias by indication.

There were some limitations in the present study. First, the study population mainly included Asian subjects. Second, types of AF and duration of each AF episode were not included in our research database. Third, the AF event definition in our study may be under-reported. We did not capture silent AF. Fourth, several confounding factors, including smoking, body mass index, and alcohol intake, were not included in our database. We also had no information on actual blood pressure control or whether the administered dose resulted in the target blood pressure. Finally, we assumed that patients took all prescribed medications. This may overestimate the actual dosage used.

## Conclusions

RAS blockers reduce the risk of new-onset AF, and are beneficial in patients aged ≥ 55 years or with a CHADS_2_ score of 1, 2, or 3. However, these therapies showed no obvious AF preventive effect in patients aged less than 55 years or with a CHADS_2_ score ≥ 4. Further studies focusing on possible biological mechanisms are needed.

## Additional Information

**How to cite this article**: Hung, C.-Y. *et al.* Age and CHADS2 Score Predict the Effectiveness of Renin-Angiotensin System Blockers on Primary Prevention of Atrial Fibrillation. *Sci. Rep.* **5**, 11442; doi: 10.1038/srep11442 (2015).

## Figures and Tables

**Table 1:** Baseline characteristics.

Variables	All patients	RAS blockers users	Non-users	*P* value
	(n = 22,324)	(n = 11,162)	(n = 11,162)	
	No.(%)	No.(%)	No.(%)	
**Age at entry, years**				
mean ± SD	65.5 ± 11.3	65.5 ± 11.1	65.5 ± 11.4	0.954
45-54	5,012 (22.5)	2,463 (22.1)	2,549 (22.8)	0.059
55-64	5,540 (24.8)	2,796 (25.1)	2,744 (24.6)	
65-74	6,792 (30.4)	3,468 (31.1)	3,324 (29.8)	
≥75	4,980 (22.3)	2,435 (21.8)	2,545 (22.8)	
**Female**	11,505 (51.5)	5,742 (51.4)	5,763 (51.6)	0.779
**Medical diseases**				
Heart failure	1,076 (4.8)	555 (5.0)	521 (4.7)	0.288
Diabetes mellitus	2,412 (10.8)	1,182 (10.6)	1,230 (11.0)	0.301
Stroke or TIA	2,341 (10.5)	1,192 (10.7)	1,149 (10.3)	0.348
Vascular disease	416 (1.9)	212 (1.9)	204 (1.8)	0.692
Thyroid disease	300 (1.3)	150 (1.3)	150 (1.3)	1.000
Valvular heart disease	247 (1.1)	132 (1.2)	115 (1.0)	0.277
Chronic lung disease	2,371 (10.6)	1,181 (10.6)	1,190 (10.7)	0.845
Chronic renal disease	551 (2.5)	279 (2.5)	272 (2.4)	0.763
**Charlson comorbidity index**				
mean ± SD	1.1 ± 1.4	1.1 ± 1.3	1.1 ± 1.5	0.794
**Medications**				
Statin	952 (4.3)	586 (5.3)	366 (3.3)	<0.001
Aspirin	3,710 (16.6)	2,150 (19.3)	1,560 (14.0)	<0.001
Warfarin	84 (0.4)	49 (0.4)	35 (0.3)	0.126
Alpha-blocker	1,534 (6.9)	885 (7.9)	649 (5.8)	<0.001
Beta-blocker	7,351 (32.9)	3,905 (35.0)	3,446 (30.9)	<0.001
Calcium channel blocker	10,164 (45.5)	5,570 (49.9)	4,594 (41.2)	<0.001
Diuretics	5,251 (23.5)	3,009 (27.0)	2,242 (20.1)	<0.001
Antiarrhythmic agent	81 (0.4)	39 (0.4)	42 (0.4)	0.738
Digoxin	278 (1.3)	158 (1.4)	120 (1.1)	0.022
**CHADS**_**2**_ **score**				
mean ± SD	1.6 ± 0.9	1.6 ± 0.9	1.6 ± 0.9	0.778
score =1	13,855 (62.1)	6,909 (61.9)	6,946 (62.2)	0.597
score =2	5,239 (23.5)	2,659 (23.8)	2,580 (23.1)	
score =3	2,010 (9.0)	989 (8.9)	1,021 (9.2)	
score ≥4	1,220 (5.5)	605 (5.4)	615 (5.5)	
**CHA**_**2**_**DS**_**2**_**VASc score**				
mean ± SD	2.6 ± 1.3	2.6 ± 1.3	2.6 ± 1.3	0.988
score =1	3,989 (17.9)	2,050 (18.4)	1,939 (17.4)	0.007
score =2	7,817 (35.0)	3,790 (34.0)	4,027 (36.1)	
score =3	5,327 (23.9)	2,711 (24.3)	2,616 (23.4)	
score ≥4	5,191 (23.3)	2,611 (23.4)	2,580 (23.1)	

**Table 2:** Dose relation analysis for new-onset AF.

Variables	No. of patients	No. of patients with AF	Incidence rate (per 1000 person-yrs)	Adjusted HR	(95% CI)	*P* **value**
Non-users (<28 days)	11,162	782	9.6			
RAS blockers users	11,162	693	7.2	0.64	(0.58-0.71)	<0.001
28-365 days	2,626	175	8.8	0.77	(0.65-0.90)	0.002
>365 days	8,536	518	6.8	0.60	(0.54-0.67)	<0.001
pure ACEIs users	2,744	155	7.5	0.80	(0.67-0.94)	0.007
pure ARBs users	1,747	84	5.4	0.64	(0.51-0.80)	<0.001
mixed users	6,671	454	7.6	0.77	(0.69-0.86)	<0.001
